# GenAI Creativity in Narrative Tasks: Exploring New Forms of Creativity

**DOI:** 10.3390/jintelligence12120125

**Published:** 2024-12-06

**Authors:** Florent Vinchon, Valentin Gironnay, Todd Lubart

**Affiliations:** Laboratoire de Psychologie et d’Ergonomie Appliquée (LaPEA), Université Paris Cité and Univ Gustave Eiffel, F-92100 Boulogne-Billancourt, France; valentin.gironnay@etu.u-paris.fr (V.G.); todd.lubart@u-paris.fr (T.L.)

**Keywords:** generative artificial intelligence, creativity, EPoC, assessment

## Abstract

This study examined generative artificial intelligences (GenAIs), as popularized by ChatGPT, in standardized creativity tests. Benchmarking GenAI against human performance, the results showed that ChatGPT demonstrated remarkable fluency in content generation, though the creative output was average. The random nature of AI creativity and the dependency on the richness of the training database require a reassessment of traditional creativity metrics, especially for AI. Our findings highlight the integral role humans play in guiding AI to foster genuine originality, suggesting the need for future research in human–AI co-creation and the development of robust AI creativity measurement mechanisms.

## 1. Introduction

### 1.1. Creativity

Creativity is defined as the ability to produce an idea, a work that is both new (original and/or uncommon) and adapted to the situation (to respond to a problem) (Lubart 1994; Runco and Jaeger 2012; Sternberg and Lubart 1995, 1998). This definition and the concepts framing creativity are subject to debate and the nature of creative behavior should be examined (Bonetto and Arciszewski 2023; Niu and Sternberg 2006; Runco and Jaeger 2012). Creativity is also seen as a capacity that is particularly characteristic of humans by many authors (Abraham 2016; Gabora 2018; Runco 2007; Sadeghi and Ofoghi 2011), linking the notion of “intention” to that of creativity or linking creativity to a genetic and neurodevelopmental context (Zaidel 2014; Zwir et al. 2021). In this study, we took the position that while AI can generate novel content, it may not fully align with traditional definitions of creativity, as it lacks the intentionality and socio-cultural context that human creativity requires (Glaveanu and de Saint-Laurent 2023). As the study of creativity was metaphorically seen as a trip across the seven seas (Cs) by Lubart (2017), we conceptualize AI creativity as uncharted territory in the creativity field.

### 1.2. AI Creativity

According to this framework, it seems difficult to consider anything other than something that is alive (animals and humans) as “creative” (Kaufman et al. 2011). Yet, with the public release of ChatGPT in 2023, many artificial intelligence developers have claimed that their tools are creative (Bard 2023; Gemini 2024; Introducing Claude 2023; LLAMA 2023; OpenAI 2023). When asked directly via prompts, these AI systems acknowledge that they are indeed AI, but will still say that they can do creative work. Artificial intelligence is defined as a system that can perform tasks that require human intelligence (Monett et al. 2020). AIs aim to replicate, or at least approximate, human cognitive functions such as perception, reasoning, problem-solving, and decision-making abilities (Veselovsky et al. 2021). They can be refined to accomplish tasks in various professional domains where their purpose might be different, for example, in music (Civit et al. 2022), manufacturing (Hou et al. 2021), therapy (Bhosale 2019), human resources (Charlwood and Guenole 2022), health (Boillat et al. 2022; Chartrand et al. 2017; Miotto et al. 2018), etc. AIs are a set of systems that can be differentiated from traditional programmed tools that can adapt to their environment using the (limited) resources to which they have access (Wang 2019). GenAIs are a family of AIs that generate content from already existing information (Muller et al. 2022; Sbai et al. 2019). AIs such as ChatGPT, Bard, Gemini, LLAMA, and Claude are LLMs (Large Language Models), which, thanks to a textual imputation (called a prompt), will give a (textual) answer by seeking to statistically predict which words come one after the other according to the given input (Lee 2023).

Because this is a series of probabilities, it is never exactly possible to predict the outcome of what will be provided, therefore, the content is “new” in its most statistic form each time. Whether it is the word order or the presentation of the idea, even if it is taken from somewhere else, the “new” character is still there. This is why Cardoso claimed in 2009 that AIs could be creative, as the new yet relevant generated content allows creative outputs to appear (Cardoso et al. 2009). But, as some other researchers point out, because GenAIs are not sentient and given the distinction between “generating” content and “being creative”, these systems therefore lack the socio-cultural construct needed to appreciate one’s creativity (Glaveanu and de Saint-Laurent 2023). Some theoretical connections can be observed with the “Blind Variation and Selective Retention” (BSVR) framework (Simonton 2023). As AI relies on vast datasets, these systems can generate ideas and propositions, but they lack the ability to assess the significance or adaptability of these ideas (Brandt 2023). It should also be noted that as stated by some authors (e.g., Doshi and Hauser 2024), even if AI is useful for enhancing “individual” creativity, and even with the massive database that they are made of, AI tends to reduce the collective novelty and diversity of the novel content. Whereas prior studies have demonstrated that generative AIs can produce content that mimics human creativity (Shimek 2023; Stevenson et al. 2022), our study sought to explore whether these outputs, when measured by standardized creativity tests, truly reflect creative potential or merely an advanced form of pattern recognition.

It has long been recognized that AIs can find new solutions to problems and provide new perspectives using their own knowledge. This observation led to the creation of the field of “computational creativity” (CC). This field focuses on the output of computational devices (computers) and the perceived creativity of this output (Colton 2008). CC research lies at the crossroads of AI, cognitive science, and social anthropology and employs an algorithmic point of view on how we as humans act in creative ways. This discipline defends AIs as co-creators or even creators in their own right but dependent on human commands (Veale and Cardoso 2019).

With the arrival of content-creating AIs among the public, we are hearing more and more cases of AIs “creating” (or co-creating) works. These range from children’s stories (Popli 2022), digital art (Roose 2022), music featuring the voices of stars (Savage 2023), or even finishing famous musical masterpieces (Elgammal 2021) to the creation of an AI art gallery in Amsterdam (Katanich 2023). The potential use of AIs is even a cause for concern, as illustrated by the strike by Hollywood screenwriters and actors, with the former fearing that their jobs will disappear, the latter that their images will be stolen and used without their consent (Beckett and Paul 2023). Other artistic and creative fields around the world are affected, such as the mistrust of the use of generative AIs in dubbing (Conradsson 2024). These various examples show that the ability to create good content is increasingly present in our society, essentially thanks to GenAI (as is the increase in deep fakes), which stem from the same technologies but sometimes with negative side effects (Hsu 2023; Murphy et al. 2023).

From a more academic point of view, some studies have already focused on the creative capabilities of AIs (Messingschlager and Appel 2022). AIs are indeed capable of generating creative products and are also able to perform creativity tasks It is thus possible to administer tasks to AIs (Bellemare-Pepin et al. 2024; Guzik et al. 2023; Hubert et al. 2024; Orwig et al. 2024; Stevenson et al. 2022), and, in theory, to measure the creative potential that these AIs have. Some of Guzik’s results indicate that ChatGPT in its GPT4 version passed the verbal Torrance Tests of Creative Thinking (TTCT) and has abilities to match those of the top 1% of the general human population in fluency and the top 3% in flexibility. In Orwig’s work, humans, GPT3, and GPT4 were used to generate multiple small creative stories (five sentences in length) and although this was an impressive result, it should be noted that because of how GenAIs are made, its fluency is systematically higher than that of humans (Habib et al. 2024; Hubert et al. 2024). As stated by Runco (2023), GenAI can only produce artefacts that would go under a framework called “Artificial Creativity”. We still feel that it is important to examine GenAI in other circumstances, to enrich, or qualify, its work.

Lubart (2017) proposed a comprehensive framework called the 7C framework, which integrates and reviews all themes explored in the creativity literature. The 7C framework consists of seven facets, namely Creators, Creating, Collaborations, Contexts, Creations, Consumption, and Curricula, which is inspired by the metaphor of the seven seas. This metaphor, derived from mythological elements, is intended to describe the different aspects of creativity that can be studied. In this context, we refer to the concept of AI creativity as “AI Land”, an unexplored territory where creativity is said to abound. However, few researchers or professionals have focused on this area. The objective of this study was to provide an overview of the findings, discoveries, and recommendations for future research and practice related to AI creativity. Specifically, this study focused on the “Creations” aspect of AI Land.

### 1.3. Evaluation of Potential Creativity (EPoC)

In order to enrich this literature and to more broadly study the potential scores obtained by GenAIs on creativity tasks, we focused on studying standardized verbal creativity tasks in two creative processes. In this context, the EPoC battery (Evaluation of Potential Creativity (Lubart et al. 2011)) is well suited for this task. EPoC uses the consensual definition of creativity, that is “creativity is the action of producing something original, new and that is relevant to the situation” (Runco and Jaeger 2012). It approaches this definition using multiple assessments techniques and using objective indicators. This is a multidimensional test used with children and adolescents that measures two fundamental creative processes. The first is divergent-exploratory thinking, which refers to a process that generates multiple possible solutions in the context of problem solving. This mode of thinking includes flexibility, divergent thinking, and selective encoding and relies on conative aspects such as openness to new experiences and intrinsic motivation (Barbot et al. 2016). The second is integrative convergent thinking, making it possible to combine, integrate, and synthesize elements, therefore involving skills of association, comparison, combination, and conative elements such as tolerance of ambiguity, risk taking, and motivation to complete a task (Barbot et al. 2011, 2016).

The EPoC verbal battery is designed to be implemented in two sessions. The first consists of three tests, one of which is a warm-up Alternative Usage Test (AUT), followed by a verbal test of each of the creative processes mentioned above. In the exploratory divergent thinking tasks, after reading the beginning or ending of a story, the participants are asked to write as many story endings (or story beginnings) as possible. In the integrative convergent thinking task, the test taker is asked to write an elaborated creative story with a specific title or with specific characters (Barbot et al. 2016). During the second session, the participants complete further exploratory divergent thinking tasks and integrative convergent tasks. These different tasks, after having been evaluated in a standardized way according to the norms of the EPoC manual, lead to composite “Divergent Verbal” (DV) and “Integrative Verbal” (IV) scores. In humans, the analysis of EPoC results reveals multiple, rather independent, creativity scores between the dimensions assessed (inter-correlation index ranging from 0.11 to 0.47, mean = 0.24 (Lubart et al. 2011)). The EPoC battery exists in multiple languages (ICIE 2011) and has been used in numerous studies since 2011. It was notably used as a pre- and post-test to see the effects of creativity-focused pedagogy in an OECD study involving ten countries (Lancrin 2020).

In the current study, we explored the creative potential of ChatGPT. At the start of the research phase, we chose the ChatGPT^1^ platform in its “Plus” subscription package form, which gives access to a chatbot powered using Generative Pre-trained Transformer 4 (GPT4) developed by OpenAI. The advantage of this model is that it is not only the best known in terms of GenAI today (with the fastest user adoption recorded), but also one of the best at the time of the data collection (Saha 2023; Touvron et al. 2023). Using ChatGPT also gave us a stable platform, offering access to the classic GPT3.5 (Legacy) and GPT4, enabling finer-grained comparisons between the two GenAIs. Furthermore, thanks to ChatGPT’s configuration, it is possible to have a new participant each time a new discussion (i.e., a “New chat”) is started (which is recorded and potentially archived). Thus, this study compared the creative potential of ChatGPT and its two models (GPT3.5 and GPT4) in different verbal tasks in an exploratory way. We also compared the results with the norms and standards described in the EPoC manual (Lubart et al. 2011).

### 1.4. Research Questions

This study addresses the following research questions (RQ):

1. How does ChatGPT perform on standard creativity tasks, as assessed by the EPoC framework?
2. What are the strengths and limitations of ChatGPT’s creative outputs in narrative tasks?
3. To what extent can ChatGPT’s outputs be considered original and meaningful in comparison to human creativity?

## 2. Materials and Methods

### 2.1. Participants

The data came from 100 “new chat” “individuals”: 50 GPT3.5 and 50 GPT4 individuals. Data from these sources were collected via the “chat.openai.com” (known as ChatGPT) platform with the “Plus” subscription package between May and June 2024. This platform allows us to start a “new chat” that has no memory of other previous discussions. As such, it allows us to have a new data source each time we started a new chat.

### 2.2. Measures and Procedures

All ChatGPT “individuals” took the EPoC verbal test in form A (corresponding to the “basic” test without corrections and approximates a normal test) in French, as the EPoC was originally designed in this language and standardized assessment norms exist for this population, thus helping to assess the different subtests and creativity indicators during the experiment. The “individuals” began with an AUT task with the prompt “*Imagine a piece of wood that comes in different shapes and sizes. Imagine all the things you can do with it. Imagine different, interesting and original ways of using this piece of wood. Try to come up with ideas of your own, ideas that the other kids won’t come up with. You’ve got 3 min to come up with as many as you can*”. This was followed by a DV1 task with a text presenting the beginning of a story, and the task of imagining endings to the story the prompt was “*I’m going to read you the beginning of a story. Try to come up with different possible endings to this beginning of a story. Try to come up with interesting and original endings to the story, different from those other children might tell. Now, listen carefully to the beginning of my story*”. After this prompt, the participant (ChatGPT) is given the beginning of the story. The final task of this session was an IV1 task, in which the “individuals” were asked to imagine a story based on a title; the following prompt was given: “*Now you have to invent a story with the following title: (title used). Try to think of an original story, different from the one the other children might tell. You have a few minutes to think up a story entitled (title used) and then tell it to me*”. In the second session, the ChatGPT “individuals” were first asked to complete the DV2 task, in which they were asked to imagine the beginning of a story based on its ending, using a prompt like DV1. Finally, the last task they had to complete was IV2, where they had to create a story comprising three characters and the following prompt was given: “*Now you have to invent a story involving X, an Y and a Z (X, Y and Z were specified). Try to come up with an original story, different from the one the other children might tell. You have a few minutes to think of a story with a X, an Y and a Z, and then tell it to me*”. The AIs produced very long content in much less time than is indicated in the EPOC manual. However, for the sake of consistency, as would have been recommended for a human subject who still had time, the experimenters suggested “relaunch” so that ChatGPT could continue its production.

The scoring was performed by noting the number of ideas for the AUT task (fluency) and DV tasks, while measuring the number of words (elaboration). For the IV tasks, story creativity was assessed on a scale ranging from “1—low creativity” to “7—high creativity” using French norms. The 200 stories were graded using the Consensual Assessment Technique (CAT) by three experimented researchers in creativity, who have previously used the EPoC on human participants after training and their scores aligned for 10% of the stories. The remaining 90% of the stories were then evaluated independently by the researchers, enabling a measure of inter-score reliability. To obtain creativity judgments of the integrative stories from ChatGPT itself, we provided it with the instructions for judges from the EPoC manual. This training started with prompt engineering, telling ChatGPT to act as a judge (CreaScoreGPT, an AI specialized in assessing and rating creativity), what its mission was (to assess the creativity of different kind of story, and giving it the initial instructions that the participants in the IV tasks were given), and how to grade the story with precise examples and benchmarks (e.g., score of 1: minimal story (usually a single sentence that combines the elements of the title or elements provided or is off-topic)).

Although this study focused on the performance of the GPT3.5 and GPT4 models in standardized creativity tasks, we acknowledge the absence of a human comparison group. The inclusion of such a group would have enabled us to better contextualize the results obtained from the AIs by directly comparing them to human responses. However, for reasons of data availability and resources, this component was not included in this study. We recommend that future studies explore this dimension to complement the perspectives proposed here. However, the overall results are discussed in relation to EPOC standards later in the article.

### 2.3. Data Analyses

Jamovi (2.3.2.1) was used to perform descriptive and inferential analyses. We performed additional hierarchical clustering analyses on the IV task stories to identify natural groupings of similar narratives. The goal of clustering is to categorize the stories into distinct groups or ‘clusters’, where stories within each cluster are more similar to each other than to those in other clusters. This allows us to detect common patterns or themes across the AI-generated stories. We used Python’s ‘Code Interpreter’ tool with the ‘AgglomerativeClustering’ method, which is a bottom-up approach to clustering that begins by treating each story as its own cluster and then progressively merges the closest pairs of clusters. To reduce the dimensionality of the story data and make clustering more efficient, we also used the ‘Truncated SVD’ package, which simplifies the data while retaining the most important information.

To determine how many clusters best represent the data, we used two indicators. The first, the Silhouette Index, measures how well each story fits into its assigned cluster. It ranges from −1 to 1, with values closer to 1 indicating that stories are well matched to their own clusters and poorly matched to neighboring clusters, whereas values closer to −1 suggest possible misclassification. The second indicator is the Davies–Bouldin Index (DBI), which evaluates how distinct and well-separated each cluster is from the others. Lower DBI values, closer to 0, suggest that the clusters are more distinct and compact, meaning that the stories within a cluster share more common characteristics, while higher values suggest greater overlap between clusters, indicating that the stories are less differentiated.

## 3. Results

To study IV task scores, we investigated the human judges’ inter-rater reliability using Cronbach’s alpha and McDonald’s omega. These were evaluated for IV1 (α = 0.80; ω = 0.82) and IV2 (α = 0.70; ω = 0.72) and the results suggested satisfactory inter-rater reliability and that the CAT can be used for score interpretation.

### 3.1. Descriptive Analysis

In terms of descriptive statistics, we found a slightly better apparent performance (regarding fluency, elaboration for the AUT and DV, and creativity score for the IV) for GPT4 compared to GPT3.5 (Table 1). Based on an ANOVA, we note that some of these differences were significant (Table 2). There was a significant difference for the AUT (*F*(1, 68.82) = 22.06, *p* < .001), and there is a significant Levene’s test at *p* < .001, thus refuting the hypothesis of equality of variances. We therefore performed a Games-Howell post hoc test to confirm the difference between GPT3.5 and GPT4 (*M_Diff_* = −4.74; *t*(68.82) = −4.70, *p* < .001). These results indicate that GPT4 can provide more AUT ideas than GPT 3.5. For the divergence tasks, the indicator that was assessed first, fluency, showed similar significant differences (DV1 Fluency *F*(1, 96.67) = 67.96, *p* < .001 and DV2 Fluency *F*(1, 97.85) = 68.93, *p* < .001), confirming the superiority of GPT4 over GPT3.5 in the ability to generate a large number of ideas. In contrast, the second indicator, elaboration, showed no significant differences between GPT3.5 and GPT4 (DV1 Elaboration: *F*(1, 96.23) = 1.66, *p* = 0.20; DV2 Elaboration *F*(1, 97.45) = 1.85, *p* = 0.18). However, this lack of significance can be explained by the standardized methodology we used: instructions were given and a “relaunch” was performed to obtain further ideas. In fact, there is no possibility for ChatGPT to exceed a certain number of characters (2048), which seems to explain the absence of significant differences between the two. More surprisingly, there were no significant differences for IV1 (*F*(1, 93.01) = 1.68, *p* = 0.20), whereas there were significant differences for IV2 (*F*(1, 96.27) = 12.59, *p* < .001). Given that this is the first in-depth evaluation of the creative ideas provided by ChatGPT, we took a closer look at these results.

### 3.2. Qualitative Analysis

The detailed qualitative study of the stories written by GPT3.5 and 4 showed that a number of elements concerning the creative production of stories written by ChatGPT “subjects” needed to be nuanced. From a descriptive point of view, some texts were noticeably plagiarized from well-known stories, as can be seen in the following example:

“*Once upon a time, there was a curious little girl named Alice. (…) she meets a white rabbit who tells her she must find a key to return to the real world. Alice begins her quest to find the magic key. She encounters a smiling cat, a smoking caterpillar and a wicked Queen of Hearts. (…)*”

In this example, from GPT3.5 to IV1, the similarity to Lewis Caroll’s *Alice in Wonderland* is particularly striking. From the character’s name to the magic key, the smoking caterpillar, and the wicked Queen of Hearts, there are numerous elements that have been placed one after the other, in a statistical fashion, recreating *Alice in Wonderland*. Other plagiarized stories were found in Task IV1, such as C.S. Lewis’s *The Chronicles of Narnia* saga or HP Lovecraft’s *The Silver Key*. In IV2, other stories were found, such as the Russian legend of *Firebird* and the Grimm brothers’ *Golden Bird*. For such stories, when they were detected by the human judges, scores of 2 or 3 were assigned depending on how much the stories varied from the originals. On the other hand, these few examples can serve to illustrate the creativity that GPT3.5 and 4 provide. Their aura of creativity is present, but when you study the details of the content, you realize that the LLMs, which generate one word after another according to statistical probability, are likely to yield similar stories in terms of content and/or form. It is particularly important to emphasize the significance of qualitative analyses regarding the work produced by GenAI. Indeed, whereas the appearance of originality is present, and plagiarism is unintentional, there is a need for increased vigilance against the potential lack of flexibility in AI systems, specifically in their ability to seek ideas from diverse fields.

The qualitative study of the results of the IV tasks also enabled us to realize the recurrence of certain “first names” for the characters in the stories. Indeed, it seems that ChatGPT, while generating quite similar stories, often uses identical names. Table 3 shows the number of different names used by ChatGPT. There was a minimum of one name per story and a maximum of three names (corresponding to the IV2 character instructions). The “Total After Cleaning Data” column was processed so that similar names were grouped together (e.g., Max and Maxime, Thomas and Tom, Maia and Maya, etc.). It seems important to note that, depending on the IV tasks, between 18% (Max or Lucas in IV2 with GPT3.5) and 30% (Lila in IV1 with GPT3.5) of the stories had a character with the same name. Of all the stories, 8.5% (Elara and/or Rosaline and/or Lisa) had at least one character with the same name. This repetition of first names also seems characteristic of an LLM where names are statistical responses to a given input, which was standardized.

### 3.3. ChatGPT Judges

As stated, we used the “Code Interpreter” function of ChatGPT, a data analysis module released in July 2023 to assess the creativity of the ChatGPT stories. To make a parallel with the human judges, we asked three different ChatGPT “judges” (in new conversations so that they would not have any memory of their scoring) to provide creativity scores using the EPoC system. Using some prompt engineering, we transcribed the human rules of the EPOC test regarding how it should grade different kinds of stories. Convinced that it could do the job, “Code Interpreter” scored the different stories according to the human rules (Score 1 = minimal story (usually a single sentence that combines elements from the title or elements provided) or off-topic to 7 = Very original story, well-constructed with many details, very good integration of the meaning of the title or the characters imposed in the instructions) but ChatGPT was inconsistent, with each judge being not significantly correlated with the other ChatGPT judges. Indeed, its scores (IV1: *M* = 3.18, *s.d.* = 1.01; IV2: *M* = 3.32, *s.d.* = 0.96) did not correlate well with those of the experimenters, and the correlations were not significantly different from zero correlation (from *r* = −0.01 to 0.14; NS, see Table 4). ChatGPT’s inter-judge reliability showed unacceptably low results (for IV1, α = 0.21; ω = 0.49; for IV2, α = 0.11, ω = 0.45). These results are surprising considering the results of other works from Organisciak et al. (2023). The “zero-shot” prompting method advocated by Organisak is in fact more closely related to the evaluation of short sentences in English. Here, we had much longer texts, and the human instructions may be incomprehensible when broken down into tokens by the AIs. Our results vary from those of previous studies, and we highlight ChatGPT’s limited ability to judge the creativity of more complex products.

### 3.4. Correlation Matrix

A detailed look at the correlation matrix revealed that only a few correlations were significant. First, the divergence in the AUT or DV (1 and 2) Fluency was correlated moderately and positively (*r* = 0.31 to 0.33; *p* < .01), showing that when the AI generated stories, it showed an associated generative capacity. In the DV tasks, fluency correlated rather strongly and positively with elaboration (*r* = 0.45 to 0.59; *p* < .001). This means that when GPT3.5 or 4 individuals were provided with many ideas, they tended to elaborate on them. Interestingly, the two fluency tasks were correlated to each other rather strongly and positively (*r* = 0.59, *p* < .001), meaning that when one of the GPTs provides many ideas for the first task, it will tend to provide a lot of ideas for the second. The rather moderate and positive correlation between DV1 Fluency and IV2 Human Scoring (*r* = 0.31, *p* < .01) suggests that the more ideas the AI generated on this divergent task, the higher the creativity scores awarded on the convergent task. This element, although explaining 9.61% of the shared variance, nevertheless seems to have had little to do with the other results obtained in the correlation matrix and should not be interpreted further until additional studies are conducted.

### 3.5. Clustering Analysis

Looking further to see how the IV stories were generated and to try to learn more about AI creativity, we worked with the Code Interpreter python module coding to perform hierarchical clustering analyses on all the stories. We then let a human decide on the optimal number of clusters based on indices such as the Silhouette Index and the DBI. The number of clusters generated according to the task and AI model is shown in Table 5 below.

Given that the Silhouette Index ranged from 0.46 to 0.55 and the DBI ranged from 0.58 to 0.70, the number of clusters was deemed acceptable according to the norms in each of the conditions presented under the “methodology”. This “objective” indicator allowed us to see that three story types were generally present in most conditions. Each of these types of stories was then repeated a large number of times with variations, corresponding to the probabilities of the LLMs displaying words one after the other. We could then better understand why, in test IV2, there were significantly different (human) creativity scores between GPT3.5 and GPT4. Indeed, whereas the “fantastic” criterion is part of the EPoC manual’s scoring grid for creative ideas, fanciful ideas were much more frequently given by GPT4, and it was the only condition to have a fourth class showing more variety in the stories. Thus, even if the increase is not large (M_GPT3.5_ = 3.33, M_GPT4_ = 3.88) in a descriptive way, we can still argue that the increased score from GPT4 in IV2 was due to a better propensity to generate variation between the stories and with the characters.

### 3.6. Creative Potential

Finally, the multifactorial approach to creativity, assessed using the EPoC norms for the French population, allowed us to give an objective score in comparison to humans. As the EPoC is a test designed for children and teenagers, we chose to compare the scores at the maximum age possible: those of a teenager in “ninth grade” (end of French middle school). The results are presented in Table 6. The EPoC quotients can be interpreted like IQ quotients (*m* = 100), with each standard deviation from the calibration population being *s.d.* = 15. For the ninth grade, the maximum score available in the norms at those quotients is 138. The EPoC can also be used to identify individuals who would be “High Potential” Verbal Creatives if they score at least one standard deviation above the mean in the two quotients mentioned previously.

The results showed that GPT4 was better overall on the Divergent Verbal Quotient (DVQ) than GPT3.5 (*t*(98) = 2.74, *p* < .01). However, for verbal divergent thinking scores, the scoring system may need to be reviewed, as it is based on fluency and ChatGPT showed a ceiling effect when scored based on human fluency norms. GPT4 always scored 138 on this scale with *s.d.* = 0, indicating a lack of variability in scores. As mentioned above, for tasks requiring content generation, it is normal for GenAIs to outperform humans. As GenAI are better than humans and are not comparable to them, it is more relevant to study the verbal integrative quotient (IVQ). Although its score was above the average for a ninth grader (population mean = 100), the scores were below the first standard deviation, indicating that the perceived quality of creativity of the stories written by GPT3.5 and GPT4 was not particularly high. It is important to note, however, that GPT4 had a statistically higher IVQ than GPT3.5 (*t*(98) = 3.60; *p* < .001), indicating that GPT4 performs better on creative integrative tasks. The “Creative Verbal High Potential” (CVHP) should normally be assessed using the two scores from the DVQ and IVQ. Due to the DVQ ceiling effect, we focused on the IVQ and found no individuals for GPT3.5, and eight individuals for GPT4 who showed high creative verbal potential which, under the norms, had an IVQ higher than 114. However, only one GPT4 individual with an IVQ of 125 would meet the strictest inclusion criteria regarding the norms to be outside of the confidence interval to be considered to have CVHP.

## 4. Discussion

The aim of this exploratory study was to observe how a GenAI (ChatGPT) performed when faced with a standardized creativity test. This study enabled us to highlight several elements, ranging from the positive to the more negative and nuanced. In this discussion, we will examine our three research questions to discuss the essence of AI creativity, at the moment, in terms of its “creations”, which we described in the introduction as the creative AI Land.

First, ChatGPT demonstrated remarkable performance on divergent thinking tasks (RQ1), particularly in terms of fluency, where GPT4 outperformed GPT3.5 and achieved scores exceeding human norms. However, these results highlight a critical methodological challenge: the limitations of traditional creativity metrics, such as fluency, when applied to generative AI, as these measures may not fully capture the unique processes underlying AI creativity.

Second, regarding the originality and meaningfulness of outputs (RQ2), the findings revealed a dual dynamic: whereas GPT models are capable of generating novel content, this originality is often undermined by recurring patterns and over-reliance on cultural tropes, raising questions about the depth and adaptive value of AI-generated creativity.

Finally, the strengths and limitations of ChatGPT’s creative outputs (RQ3) were evident in its ability to generate vast amounts of content quickly, but also in its inability to evaluate or refine this content meaningfully, as seen in the inconsistencies between human and AI assessments of creativity. Together, these findings underscore the need for new frameworks to evaluate AI creativity, integrating measures of contextual relevance, originality, and diversity, while also considering AI as a tool for human–AI co–creation rather than an independent creator.

GenAIs have an unrivalled fluency when it comes to producing content. The ability of one of these AIs to write is far superior to what any human could do, which is evident looking at the DVQ scores. Even lower models of AI generate more ideas than humans and the EPoC norms cannot reach those. As demonstrated above, AIs can generate a huge number of ideas. As noted in the discussion, the GPT3.5 or 4 scores showed a fluency that is close to, or equal to, the maximum number of ideas observed in the French norms for ninth grade students (who completed the tasks in a paper-and-pencil format, with a 10-min time limit for each DV task) on the DVQ, demonstrating its superiority over human performance. These LLMs have no inhibitions about what they can write, apart from what they have been set up to do, and the rest is just a matter of words coming statistically one after the other. In a creative test that would only consider scores such as fluency or elaboration, LLMs, as stated by Hubert et al. (2024), appear to be far better than humans. Moreover, some of the positive correlations may be more related to questions of “time of day” and server availability or unavailability. In fact, a server that is little used (in Europe, for example, in the morning, when it is the middle of the night in USA) is going to be much more available to generate ideas. Conversely, at other times, it may be saturated and provide fewer ideas.

When put side by side, the ideas provided by ChatGPT are not particularly creative. The IVQ scores were slightly above the average performance but within the first standard deviation of the EPoC scoring system. These results qualify the “creative performance” promised by the IA developers. ChatGPT is indeed capable of generating a great deal of content, but what people find “creative” is rather limited when the AI is faced with a standardized and finely defined protocol. The problem here lies in the format of LLMs, which successively predict which word should come after the other, depending on the command given. Admittedly, the content created by ChatGPT will be unique each time and may seem creative, but once it is confronted with other productions by the same GenAI model, similar patterns will emerge.

One of the main limitations of this study is the absence of comparative data from a human group. Although the AI performance was examined using existing EPoC standards, a direct comparison with human participants would have placed the results in a broader framework. Future studies could include a comparison group made up of specific populations (e.g., students or creative professionals), enriching our understanding of the relative performance of AIs and humans in creativity tasks.

Another limitation that can be added to this article is the nature of the tasks proposed in this test. Indeed, in its norms, the EPOC does not assess the notions of “adaptability” in the standard definition of creativity (Runco and Jaeger 2012) or “selective retention” of the BVRS model (Simonton 2011, 2023, 2024) or the “evaluation phase” (Sowden et al. 2015). In other words, where humans seeking to be creative are always more or less called upon some form of adaptation of the outcome, AIs call upon their data, providing generic content that will show little adaptation (Brandt 2023). Here, these theoretical considerations are all the more apparent in qualitative analyses, where LLMs show their limitations: they have impressive stocks of data but are incapable of spontaneously picking and choosing or altering their results. Thus, another avenue for future research could be prompt-engineering techniques, as well as more advanced models “verifying the logic of their responses” (e.g., ChatGPT o1) and their creative potential.

Another important point is that, unlike humans, there appears to be no particular “pattern” or “disposition” for creativity. Instead, we observed a random pathway that can sometimes lead to creativity. Of course, the ChatGPT models that were tested are all relatively recent and have plenty of room for evolution.

It also seems important to point out that “traditional” human ways of assessing creativity of AI as a tool need to be rethought. Here, fluency is an artefact that is not really relevant, as is elaboration. It depends on the number of characters an AI is capable of transcribing into its interface; the larger the interface, the greater the possibility of having a large number of ideas appear at the same time. Nevertheless, the originality of the ideas will still be interesting to evaluate. However, this assessment of originality must be made in light of what the AI has provided in multiple iterations of the same task. The proposal is to look at the ideas and evaluate them according to their similarities. Indices of lexical and semantic proximity to a sample of ideas provided to the same question would seem relevant to use to assess the creativity of artificial intelligence systems. It should be noted that this element will surely be correlated with the richness of the database used to feed the AI. The more parameters there are, the higher the chance for more creative outputs.

As mentioned above, there was not much variety in the creative stories provided by the GPT models. Faced with a single model, the same stories often emerge. These results confirm those of Doshi and Hauser (2024), who discuss the lack of “collective” novelty in favor of an appearance of individual creativity. Worse still, they may be stories that only appear to be creative, when in fact they are plagiarized from a better-known story. It is difficult, if not impossible, for a human to ensure that an AI-generated story is truly unique. The AI is not to blame for this; it has no intentionality and does not even know that it is plagiarizing a work. It is rather because of the way these AIs have been built and the database used to develop them that this problem arises. As some books and other creative masterpieces are certain to have been used to create the database, we could raise some ethical considerations regarding how, as humans, we create content. Even with the best of our efforts, our creativity is mostly built upon the work of others. Many have seen the “Mona Lisa” or other art pieces that will unconsciously nourish how we want to imagine, and then paint a portrait in the “Renaissance style”. What is interesting here is how we, as humans, allow ourselves to draw (more or less heavily) inspiration freely from different mediums and do not consider what is “nourishing” our creative representation in terms of plagiarism. Here, we are again faced with the notion of “intent” that characterizes how we should describe AI creativity. In AI-Land, maybe plagiarism is not much of a problem and maybe we should just see how well the plagiarism is used to create something that is tweaked for use in a specific situation. It must be recognized that generative AI is producing essentially a mash-up of the human-generated primary materials in the AI training database.

In fact, we risk ending up with a situation of involuntary Plagiarism 3.0, as envisaged in one of our previous works (Vinchon et al. 2023). Future research could focus on co-cre-AI-tion, as envisaged in our previous article, where humans and AIs work together to generate a truly original, new product to address the problems of the situation.

## 5. Conclusions

This article provides critical elements for our understanding of the potential of a GenAI model (ChatGPT) in 2024. When AIs are sufficiently developed, increasingly complex tests to examine their creative potential will become more relevant. Currently, AIs possess skills that enable them to generate products that resemble “human creativity”, but which are not (as they stand) the same in the scholarly sense. We do not claim that AI, as a tool, cannot deliver work that will ultimately be truly creative, but for that, we will still have to rely on humans to give content, context, and form, based on which, an AI can generate productions. It seems important to make this point clear to recruiters and decision-makers, so as not to leave AIs unsupervised in their creative tasks and risk reducing innovation and creativity.

The various analyses provided in this article should only be seen as a means of approaching what we have called “creative AI Land”. The study of the future of the field of creativity, between humans and AI, is still in its infancy, and further research will be required that may involve passing EPoC and other tasks jointly to humans and AIs in order to study the latter’s creativity. Future studies may also focus on developing objective means of scoring AI creativity, in terms of both testing protocols and the output evaluations.

## Figures and Tables

**Table 1:** Descriptive statistics of the verbal EPoC task results from GPT3.5 and GPT4.

	Module	AUT	DV1		IV1	DV2		IV2
		Fluency	Fluency	Elaboration	Scoring	Fluency	Elaboration	Scoring
Mean	GPT3.5	28.34	15.30	1056.62	4.05	13.32	1039.34	3.32
	GPT4	33.08	21.02	1110.86	4.27	18.92	1101.52	3.88
Standard Deviation	GPT3.5	2.98	3.26	224.16	0.91	3.44	236.92	0.73
	GPT4	6.48	3.67	195.59	0.72	3.31	219.67	0.84
Minimum	GPT3.5	20	7	446	2.00	5	409	2.33
	GPT4	20	11	748	3.00	10	649	1.67
Maximum	GPT3.5	30	20	1403	6.00	20	1453	5.00
	GPT4	50	31	1512	5.67	30	1543	5.33

**Table 2:** One-way ANOVA results on the different EPoC verbal tasks.

		F	df1	df2	*p*
AUT	Fluency	22.06	1	68.82	<.001
DV1	Fluency	67.96	1	96.67	<.001
	Elaboration	1.66	1	96.23	0.200
IV1	Scoring	1.68	1	93.01	0.199
DV2	Fluency	68.93	1	97.85	<.001
	Elaboration	1.85	1	97.45	0.177
IV2	Scoring	12.59	1	96.27	<.001

**Table 3:** Characters named by ChatGPT in EPoC IV tasks.

	IV1		IV2		Total After Cleaning Data
	GPT3.5	GPT4	GPT3.5	GPT4	
Number of different names	28	42	57	63	119
Mean	2.21	1.66	1.91	1.86	2.99
SD	2.94	1.94	1.83	2.01	3.70
Most used name(s) (frequency)	Lila (15)	Elara (13)	Max and Lucas (9)	Rosaline (11)	Rosaline and Elara and Lisa (17)

**Table 4:** Correlation matrix of EPoC scores.

	AUT Fluency		DV1 Fluency		DV1 Elaboration		DV2 Fluency		DV2 Elaboration		IV1 Human Scoring		IV2 Human Scoring		IV1 GPT Scoring		IV2 GPT Scoring	
AUT Fluency	—																	
DV1 Fluency	0.33	***	—															
DV1 Elaboration	0.01		0.58	***	—													
DV2 Fluency	0.31	**	0.59	***	0.14		—											
DV2 Elaboration	−0.11		0.23	*	0.32	**	0.43	***	—									
IV1 Human Scoring	0.10		0.07		0.14		−0.05		0.12		—							
IV2 Human Scoring	0.08		0.31	**	0.20		0.04		0.08		0.11		—					
IV1 GPT Scoring	−0.08		−0.03		0.04		−0.15		−0.14		0.09		0.05		—			
IV2 GPT Scoring	0.02		0.01		0.13		−0.06		−0.01		0.05		0.10		−0.01		—	

Note. * *p* < .05, ** *p* < .01, *** *p* < .001.

**Table 5:** Hierarchical classification of EPoC IV stories.

	IV1		IV2	
	GPT3.5	GPT4	GPT3.5	GPT4
No. of Clusters	3	3	3	4
Silhouette Index	0.52	0.55	0.46	0.47
DBI	0.60	0.58	0.70	0.65

**Table 6:** Creative potential standardized quotient from EPoC.

	DVQ		IVQ	
	GPT3.5	GPT4	GPT3.5	GPT4
Min	113	138	91	97
Max	138	138	114	125
Mean	136.06	138	104.18	109.16
SD	5.01	0	6.82	7.01
Creative Verbal High Potential				
GPT3.5			>*114 IVQ*	0
GPT4			>*114 IVQ*	8

## Data Availability

Due to the private nature of the “EPoC” test, the results cannot be made public. However, a summary of the results can be requested from the authors.

## Note

1 https://chat.openai.com/ (accessed on 20 March 2023).
